# The relationship between peripheral blood mononuclear cells telomere length and diet - unexpected effect of red meat

**DOI:** 10.1186/s12937-016-0189-2

**Published:** 2016-07-14

**Authors:** Marek Kasielski, Makandjou-Ola Eusebio, Mirosława Pietruczuk, Dariusz Nowak

**Affiliations:** 1 Bases of Clinical Medicine Teaching Center, Medical University of Lodz, Kopcinskiego Street 20, 90-153 Lodz, Poland; 2 Department of Laboratory Diagnostics, II Department of Internal Medicine, Medical University of Lodz, Kopcinskiego Street 22, 90-153 Lodz, Poland; 3 Department of Clinical Physiology, Medical University of Lodz, Mazowiecka Street 6/8, 92-215 Lodz, Poland

**Keywords:** Telomere length, Diet, Red meat, Peripheral blood mononuclear cells, Real-time Polymerase Chain Reaction

## Abstract

**Background:**

Repeated nucleotide sequences combined with proteins called telomeres cover chromosome ends and dictate cells lifespan. Many factors can modify telomere length, among them are: nutrition and smoking habits, physical activities and socioeconomic status measured by education level.

The aim of the study was to determine the influence of above mentioned factors on peripheral blood mononuclear cells telomere length.

**Methods:**

Study included 28 subjects (seven male and 21 female, age 18–65 years.), smokers and non-smokers without any serious health problems in past and present. Following a basic medical examination, patients completed the food frequency questionnaire with 17 foods and beverages most common groups and gave blood for testing. PBMC telomere length were measured with qualitative real-time Polymerase Chain Reaction (rtPCR) method and expressed as a T/S ratio.

**Results:**

Among nine food types (cereal, fruits, vegetables, diary, red meat, poultry, fish, sweets and salty snacks) and eight beverages (juices, coffee, tea, mineral water, alcoholic- and sweetened carbonated beverages) only intake of red meat was related to T/S ratio. Individuals with increased consumption of red meat have had higher T/S ratio and the strongest significant differences were observed between consumer groups: “never” and “1–2 daily” (*p* = 0.02). Smoking habits, physical activity, LDL and HDL concentrations, and education level were not related to telomere length, directly or as a covariates.

**Conclusions:**

Unexpected correlation of telomere length with the frequency of consumption of red meat indicates the need for further in-depth research and may undermine some accepted concepts of adverse effects of this diet on the health status and life longevity.

## Introduction

Telomeres are special structures consisting of repeating DNA chain sequences (TTAGGG) and a complex of few proteins. They are located at the ends of chromosomes and play a role in covering the cell genome and controlling number of cell divisions. Thus, affect cell lifespan. When shortening of telomeres during cell division reaches a critical length, cellular senescence is triggered. Since cellular longevity is affected by telomere length, individuals with longer telomeres should expect higher life expectancy. Very short telomere lengths may activate different repair mechanisms e.g. unlock telomerase – enzyme that can rebuild telomere sequences or ALT (Alternative Lengthening of Telomeres), which can lead to cell immortalization and tumor growth.

Numerous factors can affect shortening and rebuilding of telomeres [1–4], but previous studies did not yield a clear answer to the question what is a relationship between telomere length and some disorders [5, 6] or life expectancy [7, 8].

Diet is a common variable that can have significant impact on human health. Compliance with dietary pyramid is required for maintaining wellbeing. At the top of the pyramid is red meat, which should be eaten with moderation, preferably two or three times a week. Red meat is a good source of high amounts of protein and vitamins, especially B_1_, B_12_, PP and easily assimilable iron. Excessive consumption of red meat is accompanied by an increased ingestion of dietary fat with low level of polyunsaturated fatty acids (PUFA), and toxic substances formed during thermal treatment of meat. It may also affect the serum lipid profile while raising LDL concentration – which is widely recognized as a risk factor for cardiovascular diseases [9]. Several studies have shown that high red meat consumption can increase the incidence of colorectal and breast cancer [10–14], and DNA damage. Greater intake of red meat can induce DNA damage and may have an impact on the “Telomere Length” (TL). Main sources of DNA damage are oxidative stress [15] and inflammation. Heme iron from meat can cause DNA damage in vitro through lipid peroxidation products [16]. Increased intake of saturated fatty acids (SFA) may induce oxidative stress and thus enhance DNA-damage [17, 18].

The aim of this 3-year prospective observational study was to determine the effect of diet, smoking habit, physical activity and education on telomere length of peripheral blood mononuclear cells (PBMC). Results of a cross-sectional analysis of baseline data are presented.

## Materials and methods

### Study population

The study included 28 individuals, (21 females and seven males). A detailed description of the study population is presented in Table 1. Inclusion criteria were: age 18–65 years, smoking habit: never smoker and current smoker, no significant abnormalities on physical examination, signed informed consent form to participate in the study. Exclusion criteria: previous or ongoing major diseases including proliferative diseases and mental health disorders, pregnancy (excluded by pregnancy urine test), running disease during the follow-up to severe or poor prognosis.

**Table 1:** Characteristics of study population

Variables	Number or Mean ± SD		
Sex	21 Female		7 Male
Smoking habit			
n	16 smokers		12 non-smokers
Pack-years	16.2 ± 18.2		
Age [years]		40.8 ± 13.8	
BMI^a^		25 ± 5	
WHR^b^		0.83 ± 0.08	
LDL^c^ [mg/dL]		119.9 ± 30.7	
HDL^d^ [mg/dL]		67.4 ± 29.8	
Daily meals [n]		3.8 ± 1.0	
Education level [n]			
primary		2	
secondary		10	
higher bachelor		9	
higher master		7	
Activity level [n]			
None		7	
Low		5	
Moderate		10	
Increased		4	
Intensive		2	

^a^ *BMI* Body Mass Index, ^b^ *WHR* Waist-Hip Ratio, ^c^ *LDL* Low-density lipoprotein, ^d^ *HDL* High-density lipoprotein

### Study design

Patients enrolled to the study, after a routine physical examination, were asked to fill out a questionnaire. This questionnaire was especially developed by the authors for the study. To simplify the filling, questions with different answers (checkbox) were used. The questionnaire consisted of three parts concerning: nutrition habits, food and beverages types and physical activity. It was filled out offhand during a visit, in the presence of a physician or nurse, for additional help. After completing the survey, anthropometric measurements were conducted and detailed information about smoking habit was obtained from smokers. Previous laboratory test results (up to 12 months before enrollment) were obtained from patients’ medical records. At the end of the visit, blood was collected to determine telomere length.

### Telomere measurement

Telomere length was assessed as a relative average telomere length (T/S ratio) by PCR according to the method described by Cawthon R.M [19]. Firstly, 9 mL of venous blood was collected into EDTA tubes. Peripheral blood mononuclear cells (PBMC) were isolated from human peripheral blood by density gradient centrifugation using Histopaque® 1077 solution (Sigma Aldrich, Saint Louis, MO) according to the manufacturer’s recommendations. Afterwards, the PBMCs were washed three times in PBS and stored at −80 °C until further analysis. DNA was isolated from PBMC using QIAamp DNA Blood Mini Kit (Qiagen) according to the manufacturer's protocol. The concentration and quality of DNA obtained were assessed by spectrophotometry (Picodrop). After collecting enough samples telomere length was assessed by quantitive real-time PCR.

The primer sequences for the amplification reaction in order to determine the length of telomeres:

TelF 5'GGTTTTTGAGGGTGAGGGTGAGGGTGAGGGTGAGGGT3'
TelR 5'TCCCGACTATCCCTATCCCTATCCCTATCCCTATCCCTA3'

The primer sequences for the amplification of the reference gene 36B4

36B4F 5'CAGCAAGTGGGAAGGTGTAATCC3’
36B4R 5'CCCATTCTATCATCAACGGGTACAA3’

The reaction was carried out in triplicate. In order to perform a standard curve, dilution series of DNA were prepared (concentration range from 0.6 ng/μL to 5 ng/μL). The real-time PCR was on a 7900 HT Fast Real-Time PCR System (Applied Biosystems).

Precise reaction conditions for PCR (primer concentration, reaction time and temperature) were determined empirically. The specificity of the PCR reaction was checked based on melting curves, obtained at the end of each PCR.

### Statistical analysis

All data are presented as mean ± standard deviation or median and range. Differences between groups with normal distribution were calculated with t-test, ANOVA and adequate post-hoc tests. Survey data not normally distributed were assessed with nonparametric tests. ANCOVA models were used to adjust potential preexisting differences e.g. the effect of age or smoking habit on telomere length. All analyses were performed using the STATISTICA (data analysis software system), version 12. StatSoft, Inc. (2014) http://www.statsoft.com.

## Results

### Diet

Results of analysis of diet survey are shown in Table 2. This survey has been limited to provide eating times of a specific food per unit of time (day or week) due to the difficulty in determining the accurate food portion size. It was assumed that subjects have eaten average portion size. Available food frequency questionnaire (FFQ) proved to be too long and complicated. The survey was constructed in a comprehensible form, easy to be filled-up by all subjects and assess the average intake of groups of products (food and drinks) on a basis of daily nutrition. The survey used quantitative research methods to identify 6 “frequency consumption groups”:

F0 never
F1 once weekly or less,
F2 once daily in 2–3 days of week,
F3 once daily in 4–6 days of week,
F4 1–2x daily (at least one meal),
F5 3–5x daily (every meal),

We found no association between telomere length and the number of meals eaten per day. Eating breakfast - important for the proper diet - turned out to be irrelevant to telomeres. Neither the beginning nor the end of the daily diet had any effect on telomeres though shown its impact on external appearance. After analysis of obtained data it was found that only red meat consumption was associated with the relative length of telomeres (T/S ratio) (Table 2).

**Table 2:** Description of the consumption of various food and drink groups

	Frequency of consumption (group)		Telomere length difference	
	Median	Range	F-test	*p* value
Food				
Cereal products	F4	F1 – F5	1.17	*0.34*
Fruits	F4	F0 – F5	0.47	*0.80*
Vegetables	F4	F1 – F4	1.25	*0.31*
Dairy products	F4	F1 – F5	0.39	*0.81*
**Red meat**	F2	F0 – F4	**3.67**	***0.02***
White meat	F2	F0 – F5	1.19	*0.34*
Fish	F1	F0 – F4	0.31	*0.86*
Sweets	F2	F0 – F5	0.24	*0.94*
Salty snacks	F1	F0 – F4	0.26	*0.90*
Drink				
Fruit juices	F2	F0 – F4	0.78	*0.55*
Coffee	F4	F0 – F5	0.48	*0.70*
Tea	F4	F1 – F5	1.53	*0.23*
Mineral water	F4	F1 – F5	0.60	*0.62*
Sweet carbonated beverages	F0	F0 – F4	2.08	*0.12*
Beer	F1	F0 – F3	0.09	*0.91*
Wine	F1	F0 – F3	0.61	*0.62*
Spirits	F1	F0 – F2	1.04	*0.37*

Statistically significant differences marked in bold

**Fig. 1:**
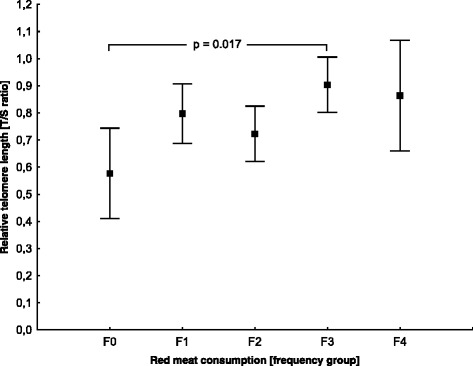
PBMC telomere length differences between red meat consumption groups. Data as mean with 95 % CI of T/S ratio, p-value of statistically significant post-hoc Tukey test, F0 - never, F1 - once weekly or less, F2 - once daily in 2–3 days of week, F3 - once daily in 4–6 days of week, F4 - 1–2x daily, F5 - 3–5x daily

The detailed relationship between consumption groups is shown in Fig. 1. Post-hoc analysis (HSD test) showed significant difference between group F0 and F3 (# p < 0.05). ANCOVA model indicated, that there was no significant interaction between age as covariate and red meat consumption and after correction of means, value F = 4.62 was still significant (*p* = 0.0078). Similarly, no effect on HDL and LDL cholesterol levels were found (F = 4.24, *p* = 0.010, F = 3.98, *p* = 0.014, respectively). The study could not confirm any relationship between types and quantities of beverages or other food groups and the length of PBMC telomeres, although other authors found such associations [20].

### Other data

Age, anthropometric data (BMI, WHR) and cholesterol levels (LDL, HDL) did not correlate with T/S ratio.

The additional aim of this study was to determine the influence of cigarette smoking on telomere length, but there was no difference between active smokers and non-smokers. In the group of smokers, daily and total burden of cigarette smoking were not correlated with T/S ratio and red meat consumption.

Physical activity, a necessary element of a healthy lifestyle was not related to telomere status. Study conditions exceed the possibilities of using more objective but time- and cost-intensive methods for determining the level of physical activity. Survey data allowed to divide participants into five groups of physical activity depending on the frequency and time of physical activity:

- None - total lack or medical contraindications for exercise
- Low - till 30 min/week
- Moderate - above 30 min/week, but less than 4x a week
- Increased - above 30 min/week and at least 4x a week
- Intensive - practicing an amateur sport with regular training

We found no differences among particular levels and T/S ratio.

Four levels of education were identified among participants (Table 1). Participants were divided into two groups – with and without higher education. PBMC telomere length T/S ratio between these groups did not differ significantly (*p* = 0.26) (Table 3).

**Table 3:** Relative telomere length (T/S ratio) of study population and compared subgroups

T/S ratio	Mean	SD	Range limits	*p* value
All	0.79	0.17	0.45–1.20	
Sex				
M	0.73	0.17	0.45–0.92	
				0.28
F	0.81	0.16	0.53–1.20	
Smoking status				
Smokers	0.77	0.22	0.45–1.08	
				0.54
Non-smokers	0.81	0.17	0.61–1.20	
Physical activity level				
None (resting)	0.71	0.14	0.55–0.89	
Low	0.83	0.10	0.71–0.95	
Moderate	0.78	0.14	0.54–0.97	0.044^a^
Increased	0.74	0.20	0.45–0.92	
Intensive	1.09	0.15	0.99–1.20	
Education level				
Lower (primary and secondary)	0.74	0.17	0.45–0.99	
				0.26
Higher	0.82	0.16	0.53–1.20	

^a^ANOVA test was significant, but no particular difference between subgroups in post-hoc HSD test

## Discussion

This study established a relationship between the relative length of telomeres in peripheral blood mononuclear cells and the frequency of eating red meat. This finding differs from those published by Lee YJ et al. [21] on the impact of dietary patterns on telomere length. This study showed that diet rich with red meat can decrease leucocyte telomere length 10 years after receiving diet data. Our participants had had blood samples collected just after filling out food frequency questionnaire. Hence we analyzed the relationship without any time shift. Our study population was a little younger (18–65 vs. 40–69 at baseline) and eating habits differ between Poland and Korea. Similarly to Lee YJ et al., a relationship was observed in colonocytes of patients who consume higher amounts of red meat [22] but not in those who ate white meat. As mentioned in the introduction, substances that enter the body along with red meat (lipids, heme iron, N-nitroso compounds) can damage the genetic material. This process is well researched in cells directly related to the digestion of red meat products, in terms particularly of carcinogenesis [23–25] can damage the genetic material. Cooking, frying and especially grilling generates substances with mutagenic activity: heterocyclic amines (HCA), polycyclic aromatic hydrocarbons (PAH), lipid peroxides, wherein the amount is dependent on the temperature of meat processing [26]. Increased consumption of processed meat correlates positively with the likelihood of breast cancer [27, 28] and negatively with leucocyte telomere length [29]. Telomere sequences may also be the site of DNA damage [15]. However, some lipid peroxidation products can reduce the risk of carcinogenesis [30]. Carnosine, a dipeptide found in red meat may have a protective effect on telomeres [31]. There is also a published study indicating the negative influence of diet devoid of meat on health status, especially increased incidence of cancer and mental health disorders [32]. This finding can support the concept of positive effects of red meat on health and is also consistent with the results of our study. The positive relationship between diet rich in red meat and the occurrence of tumors of distant organs from the digestive tract may be due to activity of red meat derivatives in the whole body. Peripheral blood mononuclear cells seem to be a good material for the analysis of the impact of red meat derivatives on the body. They are easy to isolate and count. They circulate all over the body and are exposed to the nutrient. Analysis of genetic material derived from these cells allows detection of factors that can influence changes in the genome of other tissues [33].

Our study on a small group of people managed to demonstrate the relationship between the frequency of consumption of red meat and telomere length. Although no attempt was made to estimate the amount of food products. Some studies indicate the risk of underestimating the amount of food products when using the food frequency questionnaires [34]. Micronutrients (e.g. vitamins) can be related to telomere biology, although there are large discrepancies in publications [35–39]. Our study included healthy subjects without symptoms of vitamin deficiency and who were not taking vitamin supplement. At baseline we did not measure micronutrient levels, assuming that there will not be any differences between physiological values in healthy subjects.

Age-related telomere shortening also occurs in PBMC, but there is a large variation in individual - reduction, stabilization and even increase in length [40] and it is still not fully explained [41]. Intake of food rich in small-to-medium-chain saturated fatty acids (SMSFA: milk, butter, cheese) may be associated with PBMC telomere length – inversely relative [42]. In our study we did not find such a relation (dairy products *p* = 0.81). Small amounts of SMSFA contained in the meat or used for its preparation (e.g. frying with use of butter) can be one of the TL-modifying factors mentioned above. Although high levels of LDL and HDL concentrations are associated with increased risk of cardiovascular diseases, we did not find any association between these parameters and telomere length. Similar findings were noted in other publications [43].

The study did not confirm negative effect of smoking on telomere length. This finding is probably associated with insufficient sample size. Statistical analysis also excluded the effects of smoking as a covariate modifying the TL among red meat consumers. The observation study continues and we expect changes after its completion.

Physical activity of participants did not correlate with telomere length. Two study participants with the highest physical activity had longer telomere length than others, but this difference could not be included in statistical calculations. This may suggest that only intense physical effort as opposed to mild or moderate may modify the biology of telomeres [44, 45]. Body mass index (BMI) can be associated with telomere length and there are studies in large groups of people defining the rate of TL change per BMI unit [46, 47]. We did not find any anthropometric associations – straight or reverse. Adjusting data with BMI or WHR as continuous factors did not significantly change red meat diet impact on PBMC telomere length.

Many studies indicate the relationship between TL and education level [48–50]. Less educated people are on lower incomes, often consume poor-quality foods (stale or processed) [51] containing harmful substances, which damage the genetic material. Our participants did not differ in TL among education levels, mentioned studies were based on the analysis of large populations where identifying weak dependence is easier and effect of covariates is smaller. Additionally - in contrast to our study - tests were performed among groups of people with similar age.

## Conclusions

In conclusion, our study findings are at the baseline of further observations of TL changes in response to food and behavior factors. Although we found a relatively strong relationship, it should be treated as a guide for further research on a larger group of people.

## Abbreviations

ALT, Alternative Lengthening of Telomeres; ANCOVA, analysis of covariance; ANOVA, analysis of variance; BMI, Body Mass Index; CI, coefficient interval; EDTA, ethylenediaminetetraacetic acid; FFQ, food frequency questionnaire; HCA, heterocyclic amines; HDL, high-density lipoprotein; HSD, honest significant difference; LDL, low-density lipoprotein; PAH, polycyclic aromatic hydrocarbons; PBMC, peripheral blood mononuclear cells; PUFA, polyunsaturated fatty acids; rtPCR, quantitive real-time polymerase chain reaction; SD, standard deviation; SFA, saturated fatty acids; SMSFA, small-to-medium-chain saturated fatty acids; T/S, telomere to single (copy gene); TL, telomere length; WHR, Waist-Hip Ratio
